# Mass Spectrometry-Based Differentiation of Oral Tongue Squamous Cell Carcinoma and Nontumor Regions With the SpiderMass Technology

**DOI:** 10.3389/froh.2022.827360

**Published:** 2022-03-03

**Authors:** Nina Ogrinc, Christophe Attencourt, Emilien Colin, Ahmed Boudahi, Riad Tebbakha, Michel Salzet, Sylvie Testelin, Stéphanie Dakpé, Isabelle Fournier

**Affiliations:** ^1^University of Lille, Inserm, CHU Lille, U1192 - Protéomique Réponse Inflammatoire Spectrométrie de Masse – PRISM, Lille, France; ^2^Department of Pathology, CHU Amiens-Picardie, Amiens, France; ^3^UR7516 CHIMERE, Université de Picardie Jules Verne, Amiens, France; ^4^Department of Maxillofacial Surgery, CHU Amiens-Picardie, Amiens, France; ^5^Institut Faire Faces, Amiens, France; ^6^Tumorothèque de Picardie, CHU Amiens-Picardie, Amiens, France; ^7^Institut Universitaire de France (IUF), Paris, France

**Keywords:** mass spectrometry, lipidomics, head and neck cancer, tongue squamous cell carcinoma, precision surgery, decision support, surgical margin, real-time diagnosis

## Abstract

Oral cavity cancers are the 15th most common cancer with more than 350,000 new cases and ~178,000 deaths each year. Among them, squamous cell carcinoma (SCC) accounts for more than 90% of tumors located in the oral cavity and on oropharynx. For the oral cavity SCC, the surgical resection remains the primary course of treatment. Generally, surgical margins are defined intraoperatively using visual and tactile elements. However, in 15–30% of cases, positive margins are found after histopathological examination several days postsurgery. Technologies based on mass spectrometry (MS) were recently developed to help guide surgical resection. The SpiderMass technology is designed for *in-vivo* real-time analysis under minimally invasive conditions. This instrument achieves tissue microsampling and real-time molecular analysis with the combination of a laser microprobe and a mass spectrometer. It ultimately acts as a tool to support histopathological decision-making and diagnosis. This pilot study included 14 patients treated for tongue SCC (T1 to T4) with the surgical resection as the first line of treatment. Samples were first analyzed by a pathologist to macroscopically delineate the tumor, dysplasia, and peritumoral areas. The retrospective and prospective samples were sectioned into three consecutive sections and thaw-mounted on slides for H&E staining (7 μm), SpiderMass analysis (20 μm), and matrix-assisted laser desorption ionization (MALDI) MS imaging (12 μm). The SpiderMass microprobe collected lipidometabolic profiles of the dysplasia, tumor, and peritumoral regions annotated by the pathologist. The MS spectra were then subjected to the multivariate statistical analysis. The preliminary data demonstrate that the lipidometabolic molecular profiles collected with the SpiderMass are significantly different between the tumor and peritumoral regions enabling molecular classification to be established by linear discriminant analysis (LDA). MALDI images of the different samples were submitted to segmentation for cross instrument validation and revealed additional molecular discrimination within the tumor and nontumor regions. These very promising preliminary results show the applicability of the SpiderMass to SCC of the tongue and demonstrate its interest in the surgical treatment of head and neck cancers.

## Introduction

Head and neck cancers are a heterogeneous group of tumors involving the oral cavity (OC), oropharynx, and larynx. In 2018, OC cancers accounted for over 354,000 new cases and 177,000 deaths worldwide [1]. In Europe, 128,600 new cases of lip, OC, or oropharynx cancers were diagnosed in 2020. These cancers alone were responsible for 53,900 deaths, placing them in 7th position in terms of incidence and in 9th position in terms of mortality [2]. France has some of the highest incidence and mortality rates in the old continent, particularly in men. The latest estimates show a significant overincidence of OC cancers in the north of the country, with the Hauts-de-France region being the most affected [3]. Squamous cell carcinomas (SCCs) are the most common OC cancers in over 90% of cases [4], with the (mobile) tongue (OT) as one of the most frequent sites [5]. Now, there is an increasing incidence for people with <45 years and in women [6]. Main risk factors include tobacco use, alcohol abuse, and high-risk human papillomavirus (HPV) (types 16 and 18 principally) [7]. Unfortunately, OC SCCs are often diagnosed late, sometimes at an advanced stage, even for locations that allow direct visual or tactile examination [8]. Significant progress has been made in their treatments over the last 30 years; however, they still display poor prognosis (excluding HPV-related cancers), with a 5-year survival slightly higher than 50% [9], a high risk of recurrence (50–60% of cases), and the presence of distant metastasis within 2 years of the initial diagnosis [10]. Oral tongue SCC generally behaves aggressively and has been associated with high rates of distant and lymph node metastases due to the rich lymphatic network [11]. Nowadays, the prognosis is still mainly based on the tumor, node, and metastasis (TNM) staging of the tumor [12] and the primary treatment strategy relies on the complete surgical resection [4]. The surgical margins status is one of the key factors weighing on the prognosis and defining the need for adjuvant treatments [13]. It has been shown that positive margins in OC carcinoma lead to a risk of locoregional recurrence between 16 and 20% [14] and reduce the 5-year survival by 11–15% [15]. In clinical practice, surgical margins are defined intraoperatively, using visual and tactile information, but in 15–30% of cases, positive margins are found after histopathological examination several days postsurgery [16]. It is, therefore, essential to develop new ways of assessing surgical margins directly in the operating theater in order to avoiding locoregional relapse for patients in the future. For this reason, real-time evaluation methods have been considered in the recent years [17, 18].

Several emerging technologies have paved the way for molecular guidance in the clinical setting [19]. Among them, real-time molecular profiling by mass spectrometry (MS) coupled to either surgical [20–23], laser [24–26], or liquid extraction devices [27] has demonstrated their potential to discriminate the tumor and nontumor cell phenotypes. The so-called SpiderMass technology based on water-assisted laser desorption ionization (WALDI) is designed for *in-vivo* real-time analysis under minimally invasive conditions [24, 25, 28, 29]. This instrument achieves tissue microsampling and real-time molecular analysis with the combination of a laser microprobe and a MS instrument and is, thus, ultimately a tool for histopathological decision support and surgical guidance. The system can be run intraoperatively or postsurgery as well as for retrospective analysis in pathology [30]. In the operating theater, the SpiderMass could also become an innovative surgical and imaging tool to precisely define the tumor edges and surgical margins during the excision [29, 31]. Based on the principle of machine learning, the SpiderMass instrument must initially be thought to recognize specific molecular profiles of different cell phenotypes [25, 29]. The performances were already evaluated for *ex-vivo* molecular lipid-based diagnosis of sarcomas from dog patients and then tested *in vivo* at the veterinary surgery room [29].

Due to its ability to differentiate cancer cells, the SpiderMass technology is of great interest to assess the margin status in SCC. Here we present a noninterventional prospective pilot study based on surgical excess tissue from tongue SCC resections, which aims to build classification models by machine learning from known and histologically annotated SCCs. The built models will then serve as the basis for the SpiderMass application *in vivo* during surgical management.

## Materials and Methods

### Study Design

This is a prospective, noninterventional pilot study based on surgical excess tissue from oral tongue SCC resections. This study has been approved by the ethics committee of the Amiens-Picardie University Hospital (protocol number PI2020_843_0156), performed in accordance with the ethical standards of the Declaration of Helsinki and its later amendments, and registered on ClinicalTrials.gov (NCT05104619).

Regarding the prospective collection of samples, informed consent for clinical and biological data collection and publication was obtained for each patient at the time of hospitalization before surgical intervention. Concerning the retrospective analysis of formalin-fixed paraffin-embedded (FFPE) samples, all the patients had been informed at the time of care that their standard clinical and biological data could be used for research purposes, according to the French Public Health Code and in application of the General Data Protection Regulations and none had expressed his opposition. This study is registered under the Reference Methodology 004 of the Commission Nationale de l'Informatique et des Libertés (CNIL), French Data Protection Agency.

### Participants

This study population consisted of 7 prospective patients, 18 years and older, treated between March and September 2021 in the Department of Maxillofacial Surgery at the Amiens-Picardie University Hospital (Amiens, France) for tongue SCC (±oral floor), tumor stage T1 to T4, with the surgical resection as the first line of treatment. Recurrent or metastatic diseases and patients who previously benefited from neoadjuvant chemotherapy and/or radiotherapy were excluded from this study. An additional group of seven patients with oral tongue SCC (±oral floor) was retrospectively included to obtain a total number of 14 FFPE slides from different patients, with the aim of constructing the SpiderMass databank following the established protocols. Table 1 shows the demographic and clinical data by the prospective/retrospective group and for all of the patients included in this study. Table 2 specifies the clinical and demographic data and the types of samples available for each patient of the two groups.

**Table 1:** Demographic and clinical data for the 2 groups and for all the patients.

		**Prospective**	**Retrospective**	**All patients**
		**group (*n* = 7)**	**group (*n* = 7)**	**(*n* = 14)**
Age, *median (range)*		58 (48–77)	65 (51–73)	62.5 (48–77)
Sex	Females, *n* (%)	3 (42.86)	2 (28.57)	5 (35.71)
	Males, *n* (%)	4 (57.14)	5 (71.43)	9 (64.29)
Habits	Tobacco, *n* (%)	5 (71.43)	6 (85.71)	11 (78.57)
	Alcohol, *n* (%)	4 (57.14)	4 (57.14)	8 (57.14)
Tumor site	Oral tongue only, *n* (%)	5 (71.43)	3 (42.86)	8 (57.14)
	Oral tongue and floor, *n* (%)	2 (28.57)	4 (57.14)	6 (42.86)
Overexpressed p16, *n* (%)		3 (42.86)	1 (14.29)	4 (28.57)

**Table 2:** Detailed demographic, clinical, and sample data of the patient.

**Patient number**	**Age**	**Gender**	**Tumor location**	**Tobacco consumption**	**Alcohol consumption**	**Intervention date**	**Margin status**	**P16 status**	**Fresh frozen samples**	**FFPE samples**
**Prospective group**										
SPT A	48	M	Tongue	Y	Y	11/03/2021	Clean	+	Y	Y
SPT B	52	F	Tongue and oral floor	Y	N	02/04/2021	Clean	–	Y	Y
SPT C	77	F	Tongue	N	N	28/04/2021	Clean	–	Y	Y
SPT D	58	M	Tongue	Y	Y	18/05/2021	In contact with the median and posterior borders	–	Y	Y
SPT E	73	M	Tongue	Y	Y	29/06/2021	Clean	+	Y	Y
SPT F	59	F	Tongue and oral floor	Y	Y	03/08/2021	Clean	+	Y	Y
SPT G	58	M	Tongue	N	N	29/09/2021	Clean	–	N	Y
**Retrospective group**										
6765	67	M	Tongue (history of soft palate SCC)	Y	N	04/05/2021	Clean (deep margin <1 mm)	+	N	Y
1251	51	M	Tongue and oral floor	Y	Y	23/01/2020	Clean	–	N	Y
9974	71	F	Tongue	N	N	05/08/2020	Clean	–	N	Y
11955	62	M	Tongue and oral floor	Y	Y	17/09/2020	Clean	–	N	Y
11846	65	F	Tongue and esophagus (recurrence)	Y	Y	15/09/2020	Clean	–	N	Y
13917	63	M	Tongue	Y	Y	10/09/2019	Clean	–	N	Y
11652	73	M	Tongue and oral floor	Y	N	11/09/2020	Clean	–	N	Y

*+, overexpressed P16 status; –, non-overexpressed P16 status*.

### Samples Preparation

#### Tissue Cryosections

Fresh tissue was removed from both the tumor and peritumoral areas immediately after surgery. Samples were first examined by a pathologist to macroscopically delineate the tumor, dysplasia, and nontumor areas. Representative fresh tissue fragments of 2 cm × 2 cm × 0.5 cm were selected and placed on top of a drop of water on a precooled sample disk (−20°C) in a cryomicrotome (Cryostat, Thermo Fisher Scientific CryoStar^TM^ NX70, MMFRANCE®). Three consecutive slides were then prepared: (i) 7 μm thick sections for H&E staining, (ii) 20 μm sections for MS analysis by the SpiderMass®, and (iii) 12 μm sections mounted onto a conductive glass slide coated with indium tin oxide (ITO) for imaging analysis by MS (MSI). The H&E glass slides were annotated by the expert pathologist to delineate the tumor and nontumor regions. All the mounted sections were stored in a sealed container, H&E slides at 4°C, ITO and the SpiderMass slides at −80°C at the Tumorothèque de Picardie (ISO 9001-2015) prior to their use.

#### Formalin-Fixed Paraffin-Embedded Tissue Section

Archived FFPE blocks were retrieved from the tissue bank of Picardie of the pathology department. Three μm thick FFPE tissue sections were cut by using a microtome (Thermo Fisher Scientific HM 355S automatic rotary microtome, MMFRANCE®) at room temperature. For each case, stained and nonstained slides were prepared (Matsunami TOMO® hydrophilic adhesion slides, VWR-France). For standard hematoxylin phloxine saffron (HPS) staining, we used the Tissue Tek Film™ machine (Sakura®, France). All the slides were stored at room temperature. The first 3 μm section was stained and the consecutive section was prepared for the SpiderMass analysis. The FFPE tissues for the SpiderMass analysis were manually sprayed with a glycerol/isopropyl alcohol (IPA) (8:2, v/v) solution in two successive passes by using a manual sprayer (Agilent). The syringe pump (74900 series Cole Parmer Instrument Company) was set to a 300-μl/min flow rate. The two successive passes were equal to 5 μl deposited on 1 cm^2^ as previously described [30].

### SpiderMass Analysis

The basic design of the instrument setup has been described in detail elsewhere [25, 28]. Briefly, the system is equipped with a remote fibered tunable wavelength laser system (Radiant version 1.0.1, Opotek Incorporation, USA) and pumped by the 1.064 μm radiation delivered by a Q-switched 10 ns pulse-width neodymium-doped yttrium aluminum garnet (Nd:YAG) laser (Quantel Laser, France). The IR laser microprobe is tuned at 2.94 μm to excite the most intense vibrational band of water (O-H). A 1-m biocompatible laser fiber with 450 μm inner diameter (HP fiber, Infrared Fiber Systems, USA) is connected to the exit of the optical parametric oscillator (OPO) and focused to result in 400–500 μm beam diameter. The microprobe is attached to a robotic arm for automated acquisition [31]. A Tygon® tube (Akron, USA) is used to aspirate the ablated material and is connected to a quadrupole time of flight (Q-TOF) mass spectrometer Xevo (G2-S, Q-TOF, Waters, Manchester, UK) through a rapid evaporative ionization mass spectrometry (REIMS) prototype interface [20]. The laser sampling position on the SCC samples was determined based on the histopathological annotations. The samples were randomized to avoid batch effects. The acquisition was composed of 10 laser shots at 4.0 mJ/pulse consecutively fired 3 times with a 10-s pause at random position from the annotated tumor and nontumor regions. Ten laser shots resulted in one individual MS spectrum consisting of several *m/z* values corresponding to lipid molecules. The data were acquired in positive and negative sensitivity modes with 1 s scan time and 22,000 mass resolution. An isopropanol solution of 200 pg/μl LeuEnk was used for lock mass correction. The FFPE tissue analysis was performed after application of glycerol described in sample preparation. It composed of 10 laser shots consecutively fired 3 times with a 10-s pause in the tumor and nontumor regions. No lock mass infusion was used during the FFPE tissue analysis.

### Matrix-Assisted Laser Desorption Ionization (MALDI) MSI Analysis

The fresh-frozen 12 μm mirror tissues thaw-mounted on conductive ITO slides (LaserBio Labs, Valbonne, France) were used for MALDI-MSI analysis. The sections were coated with 12 layers of Norharmane matrix [7 mg/ml in (v:v) 2:1 CHCl_3_:MeOH] deposited by the pneumatic spraying (M5 sprayer, HTX Imaging). All the data were acquired on the Bruker RapifleX MALDI Tissuetyper™ System operating at 10 kHz in reflectron mode (Bruker Daltonik GmbH) with a nominal acceleration potential of ± 20 kV. MSI data were acquired by using a 50 × 50 μm^2^ raster and a 20 × 20 μm^2^ beam scan area for dual polarity imaging [32, 33]. The calibration was performed by using red phosphorus in the 100–3,000 *m/z* range.

### Statistical Analysis

The raw SpiderMass data were imported into “Abstract Model Builder”—AMX (version 1.0 1972.0, Waters, Hungary). The classification model was built by using individual spectra (on average 3 per sample) and by subjecting them to principal component analysis (PCA) and linear discriminant analysis (LDA). The classification models were built by using a mass range of 600–1,000 *m/z* with a 0.1 binning, 1e^5^ threshold intensity, applied normalization, and background subtraction. The discriminative ions present in LDA were tentatively annotated by Alex123 with the precursor ion set with 0.05 *m/z* tolerance including the possibility to observe [M-H]^−^, [M+CH_3_COO]^−^, and [M+HCOO]^−^ ions for the negative and [M+H]^+^, [M+Na]^+^, and [M+K]^+^ for the positive ion mode [34]. The MALDI-MSI datasets were processed by using SCiLS Lab MVS 2021b (Bruker Daltonik GmbH) software. The data were total ion count (TIC) normalized by using weak denoising deterministic installation for both the polarities. Automatic peak picking was performed by using the default segmentation pipeline. The spatial segmentation algorithm was applied individually to each tissue to display intratissue heterogeneity [35]. The spatial segmentation groups together all pixels with similar spectra into colored, expandable clusters called segments. All the pixels corresponding to the same cluster are than represented by the same color. The segmentation generally displays a molecular profile (several m/z values and each m/z corresponds to one molecule) associated with each cluster. The segmentation is represented as a dendrogram and the branches of the dendrogram are calculated by the correlation distances between each cluster. The smaller the distance, the closer the molecular profiles are between each segment.

## Results

### Classification of SCC From FFPE Samples

**Figure 1:**
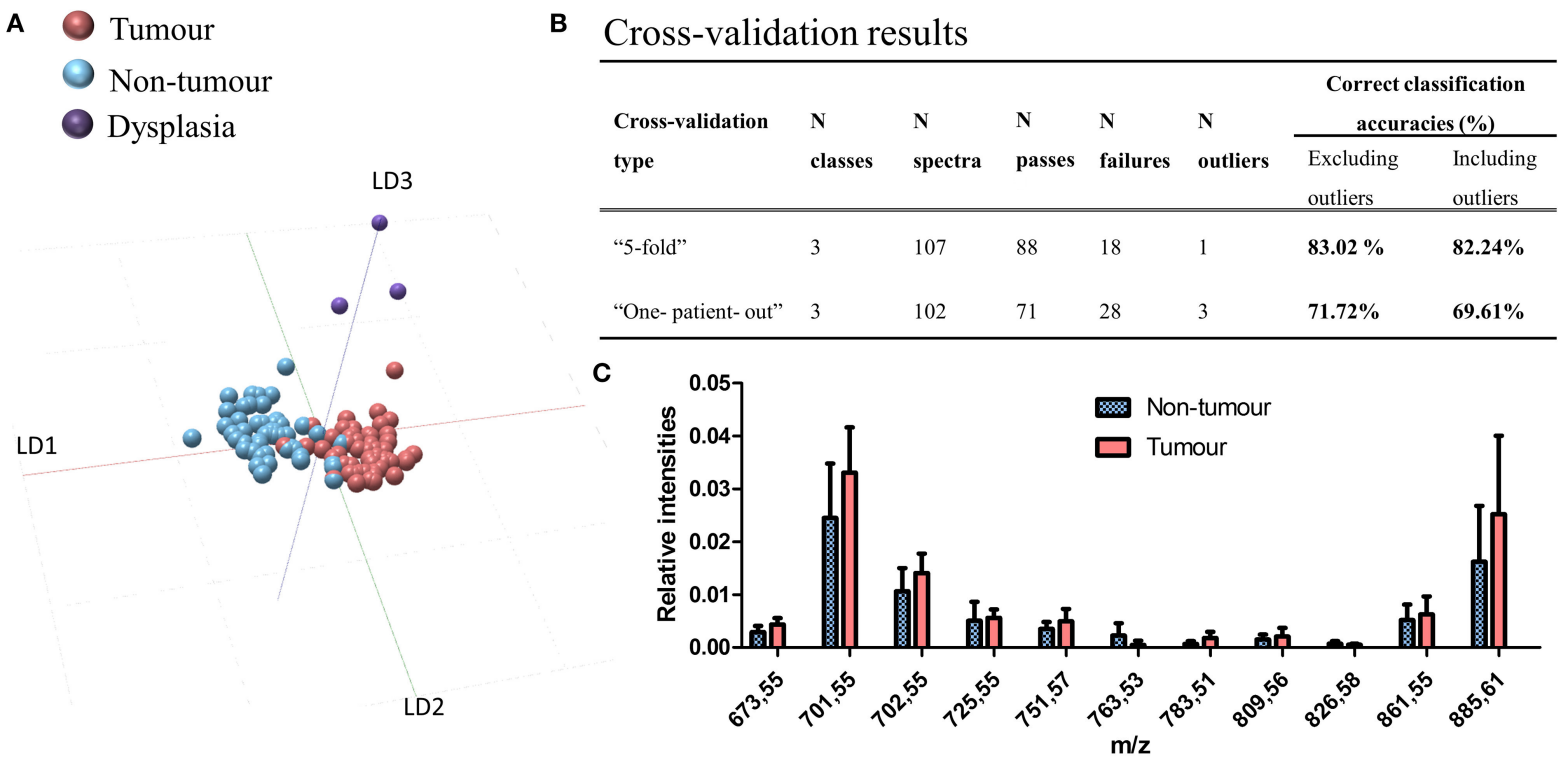
Formalin-fixed paraffin-embedded (FFPE) tissue analysis by the SpiderMass. **(A)** Principal component analysis-linear discriminant analysis (PCA-LDA) classification model based on the tumor, nontumor, and dysplasia annotated regions of the tissue. **(B)** Cross-validation results. Cross-validation results of the 3-class model with model type, cross-validation type, N of classes, N spectra, N of passes as well as N of failures, N outliers, and % of correct classification accuracies after cross-validation. **(C)** Relative intensity boxplots of the selected peaks showing a discrimination in LD1 for the nontumor and tumor regions, respectively. The intensities were normalized to the total ion chromatogram and represented as boxplots with the Tukey method whisker definition.

The initial MS tissue-type databank was created by using a pilot cohort of 14 oral SCC FFPE samples (7 prospective and 7 retrospective patients, 14 samples, 47 nontumor, 55 tumor, and 3 dysplasia spectra). After postoperative histopathological annotations, the samples were processed following optimized procedures for FFPE databanks. The SpiderMass analysis was realized in negative ion mode where lipidomic profiles reached sufficient intensities (1e^4^) to build classification models. The sampling points consisted of the nontumor, SCC, and dysplasia regions in clean margins representative of true pathological changes. The oral dysplasia was graded by using 3 tiers scheme proposed by the WHO head and neck tumor classification [36]. Patient demographics and samples are shown in Tables 1, 2, respectively. We first investigated, if the lipidomolecular profiles could discriminate between the nontumor, the SCC, and the dysplasia areas. The molecular profiles were subjected to PCA and LDA. The results are shown in Figure 1A. The first and the second components separate the nontumor and tumor tissues, while the third component separates the dysplasia. The model gives cross-validation accuracies of 83.2% by using “5-fold” and 71.7% by using “leave-one-patient-out” methods (Figure 1B) for 14 patients. The most discriminative lipid peaks and their relative abundance in the tumor and nontumor tissues are shown in Figure 1C. The discriminatory peaks are mainly composed of phosphatidic acid (PA), phosphatidylinositol (PI), diacylglycerols (DAGs), and triacylglycerols (TAGs) lipid species (Supplementary Table S1).

### Prospective *ex-vivo* Databank for Intraoperative Intervention

**Figure 2:**
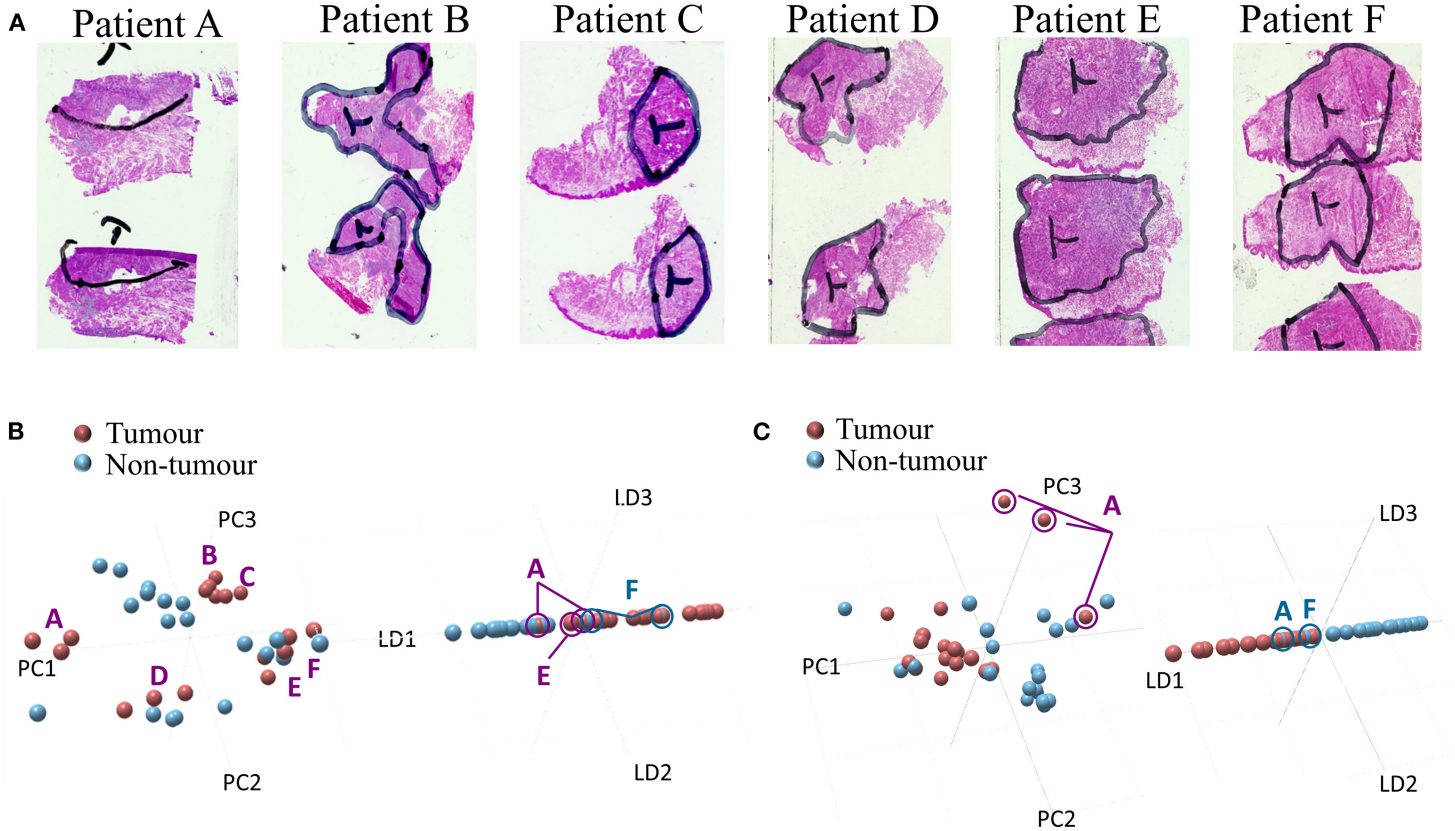
The prospective cohort SpiderMass analysis and classification models. **(A)** Examples of optical images of histopathological stains and annotations from fresh-frozen squamous cell carcinoma (SCC) samples. The annotations for patients A, B, C, D, E, and F delineate between the tumor and nontumor regions analyzed by the SpiderMass. Random spots were analyzed from each region. The multivariate statistical analysis of mass spectrometry (MS) data from the SCC samples in **(B)** positive and **(C)** negative ion mode; tumor (red) and nontumor (blue). PCA plot of 2 tissue types (left) and LDA model representation with a two-class scheme (right). The markers A-F indicate each patient.

The SCC prospective study was composed of 7 patients (7 FFPE samples and 6 fresh-frozen samples) with varying stages and surgical resection as the first line of treatment. The number of patients was limited by the number of surgical interventions in 2021. The patient demographic information is shown in Table 1 and the collected samples information is shown in Table 2. The fresh-frozen lipidomic databank, following standard intraoperative procedures, was constructed by classifying tissues according to the histopathological annotations of the nontumor (*n* = 18–20 spectra) and tumor (*n* = 18 spectra) regions (Figure 2A). The results are shown in Figure 2B for the positive and Figure 2C for negative ion modes. Both polarities were chosen in order to capture different lipidomic species present in the tissue regions. The PCAs show no particular variance between the nontumor and tumor tissues due to intra- and interpatient variability. Patients A and D show a high degree of variance in the data. On the other hand, the patients B and C group together as well as patients E and F in positive ion mode. The PCA analysis in negative ion mode, however, shows a discrimination of the nontumor and tumor tissues along the first principal component (PC1) with few exceptions. Patient A, for example, seems to exhibit profiles closer to the nontumor tissues, while several nontumor tissues exhibit profiles closer to tumor tissue. Further, PCA-LDA revealed a discrimination between the nontumor and tumor tissues in both the positive and negative ion modes. The mass loadings plots are shown in Supplementary Figure S2. Lipids contributing to the discrimination mostly correspond to a mixture of phosphatidylcholines (PCs) and phosphatidylethanolamines (PEs) in positive ion mode and PA, phosphatidylserines (PSs), and PI in negative ion mode. The tentative annotations are shown in Supplementary Table S2. Similarly, to PCA, the PCA-LDA revealed several tumor spectra to be classified as nontumor and vice versa. This is particularly true for patients A and F.

### Delineation of the Tumor and Nontumor Tissues Heterogeneity

**Figure 3:**
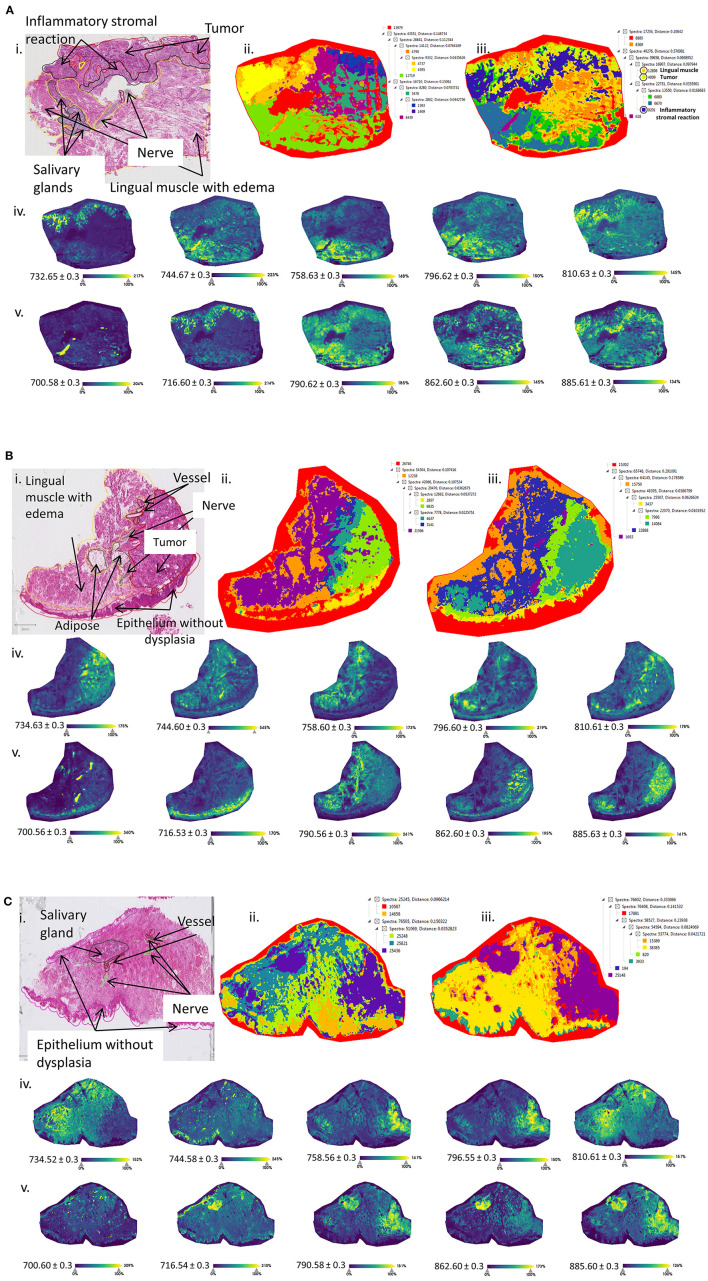
Optical images and MS imaging data of the prospective cohort. Matrix-assisted laser desorption ionization at 50 μm spatial resolutions was used to measure sections in both the positive and negative ion modes. All the data were subjected to the bisecting K-means segmentation. Each part of the figure presents the: (i) detailed histopathological annotations, (ii) image segmentation and cluster tree in positive ion mode, (iii) image segmentation and the cluster tree in negative ion mode, (iv) selected ion images in positive ion mode *m/z* ± 0.3 and (v) selected ion images in negative ion mode *m/z* ± 0.3 for **(A)** patient A, **(B)** patient C and **(C)** patient F. All of the selected ion images were found discriminative with the prior SpiderMass analysis.

To further investigate the results and appropriateness of the prospective classification models based on just the tumor and nontumor tissues, we examined the intratissue heterogeneity of each patient. The analyses were conducted on 4 patient samples [patients A, C, D (data not shown), and F]. First, a more detailed histopathological annotation was performed by the pathologist delineating regions such as nerves, salivary glands, lingual muscle with edema, epithelium, and inflammatory reactions. Second, MALDI-MSI in dual polarity mode was performed on consecutive mirror tissue sections initially analyzed by the SpiderMass. The results from patients A, C, and F are shown in Figures 3A–C. Each figure presents the: (i) histopathological annotations, (ii) image segmentation in positive ion mode, (iii) image segmentation in negative ion mode, (iv) selected ion images in positive ion mode, and (v) selected ion images in negative ion mode. All of the selected ion images were also found discriminative by the SpiderMass analysis. Histopathological annotation and segmentation analysis of patient A (Figure 3A) revealed an additional inflammatory reaction under the tumor region previously annotated as tumor. Particularly, in negative ion mode, the segmentation (Figure 3Aiii) highlights the dark blue region corresponding to the inflammatory reaction with a different molecular profile. The MALDI image segmentation also reveals the nerves (purple) and the lingual muscle with edema (orange). The latter appears to cluster closer to the tumor region. The selected ion images *m/z* 732.65, 744.67, 758.60, 796.60, and 810.62 ± 0.3 in positive ion mode and *m/z* 700.57, 716.60, 790.62, 862.60, and 885.60 ± 0.3 in negative ion mode also display a correlation with the histopathological annotations. For example, the ion at *m/z* 700.57 [PE (O-34:2)-H]^−^ or [HexCer 34:0;2-H]^−^ is highly distributed in the nerves, while the ion at *m/z* 885.60 [PI (20:4_18:0)-H]^−^ is also distributed in the inflammatory reaction. The same ion was found to be discriminative in peritumoral region with the SpiderMass analysis. Patient C includes several additional structures such as the lingual muscle with edema, several vessels, nerves, adipose tissue, and epithelium without dysplasia (Figure 3Bi). Equally, the structures are observed in both the positive and negative ion modes (Figures 3Bii, iii). The tumor region is very well defined (green) as well as the nerves, epithelium, and the lingual muscle with edema. In this case, there is a larger distance between the tumor tissue and the lingual muscle. The selected ion images in positive ion mode correlate with the SpiderMass data (Figure 3Biv); however, the ion at *m/z* 700.57 is distributed in the nerves and the ion at *m/z* 716.60 [PE (34:1)-H]^−^ in the epithelium. Interestingly, the ion at *m/z* 885.60 [PI (20:4_18:0)-H]^−^ is distributed not only in the lingual muscle, but also in the tumor region (Figure 3Bv). Patient F represents a difficult case because the tumor is characterized by very low cellularity, diffused infiltration, and inflammatory stromal reaction. The annotations indicate the presence of a salivary gland, nerves, vessels, and epithelium without dysplasia (Figure 3Ci). The segmentations well delineate the salivary gland and the undefined region on the right-hand side, previously indicated as the nontumor region (Figures 3Bii, iii). In the positive ion mode, several ions are shown to be distributed in the tumor region (Figure 3Civ), while in the negative ion mode (Figure 3Cv), the ions are distributed in the salivary gland and the peritumoral region.

## Discussion

We have successfully created the SpiderMass histolipidomic databanks following the line of noninterventional intraoperative and postoperative analysis of SCC. This preliminary study was focused on the discrimination and building classification models of the tumor, nontumor, and dysplasia regions of clear margins. This study included several patients with different genders, ages, and habits including tobacco and alcohol consumption. Generally, the surgical margins are defined in consultation of intraoperative frozen sections. This is an examination performed while the patient is under anesthesia on the operating table. After gross examination of the fresh tissue, frozen sections are stained (H&E) and their morphology is examined by microscopy. The results are then sent back as soon as possible to the surgeon and will be used to make a decision on the surgical procedure to be conducted. The initial diagnosis is confirmed by the histopathological examination of the FFPE samples postsurgery within several days. We first evaluated the possibility to construct the SpiderMass databank from the FFPE samples with 14 patients following the established protocols [30]. The construction of the database revealed discriminative lipid profiles between the nontumor and tumor regions. The SCC models gave good classification accuracies 83.0% with “5-fold” cross-validation method and 71.7% with the “leaving-one-patient-out” similar to what has been previously observed with the FFPE human sarcoma samples for the underrepresented number of patients [30]. In the case of SCC, however, the analysis was performed in the negative ion mode. This strongly coincides with the fact that the detection of different lipid species in the FFPE tissues depend on the tissue types of clinical samples [30, 37, 38]. Nonetheless, the detected lipids, mainly PAs and PIs, were also previously found to be preserved in formalin-fixed tissues [39]. So far, the classification accuracy is sufficient considering the size of this pilot cohort (14 patients), but we expect further improvement by increasing the number of patients included in this study. In addition, this larger study will also allow us to further investigate the molecular distinctions between negative margins and peritumoral areas as well as positive and negative margins. In parallel to the FFPE databank, a prospective frozen tissue databank was created from tongue SCC resections from 6 patients. The histopathological annotations first delineated the tumor and nontumor regions. In this case, the lipidomic profiles well separated the two regions, but with several exceptions. The tumor region of patient A was, for example, shown to be closer to the nontumor region and vice versa. A similar trend was found with patient F. In contrast to the SpiderMass FFPE analysis, the data show larger variability between patients. This could suggest that fragments from the FFPE operative specimens used retrospectively are often more representative tumor samples compared to frozen tissue prepared macroscopically by the pathologist from excised tissue. Some studies have shown that this variability even depends on the type of tissue analyzed [40, 41]. In addition, several lipid subclasses are not preserved during the formalin fixation and paraffin embedding process [30, 39, 42]. Further histopathological investigation of frozen sections of patients A, C, and F revealed the presence of an inflammatory stromal reaction and other regions within the tumor. The inflammatory stromal reaction in patient A also showed specific molecular signatures by MALDI-MSI. Moreover, the lingual muscle with edema seems to display a closer molecular profile to the tumor region based on segmentation clusters. Due to the size of the SpiderMass microprobe beam (400–500 μm), more than one type of cells is analyzed by increasing the probability to include several distinct regions and cell type within the MS spectrum. Also, the frozen tissues were analyzed by using a gross annotation by the pathologist similar to expected extemporaneous conditions. This clearly demonstrates that such annotations are insufficient for SCC and more details are required to obtain a high learning specificity and sensitivity of the SpiderMass system. Patient F also revealed the distribution of certain *m/z* such as the 885.60 [PI (20:4_18:0)-H]^−^ present in both the nontumor and salivary gland in the tumor region. The aforementioned reasons would explain why some nontumor tissues were classified as tumor and why some tumor tissues were classified as nontumor. All together, the results reflect the high sensitivity of the SpiderMass analysis without prior detailed annotation.

Mass spectrometry is an attractive technology for the purpose of margin evaluation due to the specificity of the molecular information retrieved from cell phenotypes. Therefore, several MS systems have already been evaluated for the intraoperative investigation of surgical margins or classification purposes [23, 27, 43]. For example, *in-situ* desorption electrospray ionization (DESI) was used *ex vivo* to determine the safe surgical resection margin in OSCC [44] and for the evaluation of translation from *ex-vivo* studies to *in vivo* for brain and gastrointestinal tumors [45–47]. In the latter, during the gastric surgery, consecutive sections of margins were analyzed by DESI-MSI and classified by an automated Lasso system (multiclass-logistic regression with L1 penalty). The results of the classification were in close agreement with the histopathological annotations, but with few exceptions. The exceptions were for a distal margin of the patient with first gastric cancer, for a proximal margin of the patient with second gastric cancer, and for the proximal and distal margins of the patient with third gastric cancer [47]. The same Lasso method was used for rapid diagnosis and tumor margin assessment of pancreatic tumors with the MasSpec Pen. Three distinct regions on large surfaces of tumors were classified against the pancreatic ductal adenocarcinoma (PADC) and normal tissue. While in high agreement with the histopathological evaluation, difficulties remained for the evaluation of cases with low infiltration of cancer cells [48]. These examples clearly demonstrate the need for MS analysis in the assessment of surgical margins, but even if all the MS-based technologies demonstrate strong classification performances, improvements are still necessary to enable complete characterization including the percentage of cancer cells present at a specific location.

In conclusion, we have shown a MS-based technique to characterize the dysplasia, nontumor, and tumor regions of SCC clean margins throughout the noninterventional intraoperative and postoperative examination. This pilot study also suggests that the SpiderMass technology is sensitive and specific enough to detect subtle changes within the tumor and nontumor tissues as well. The built models serve as a promising basis for the SpiderMass to be used *in vivo* during surgical management of SCC in the OC.

## Data Availability Statement

The raw data supporting the conclusions of this article will be made available by the authors, without undue reservation.

## Ethics Statement

The studies involving human participants were reviewed and approved by Ethical Committee of the Amiens-Picardie University Hospital (protocol number PI2020_843_0156), performed in accordance with the ethical standards of the Declaration of Helsinki and its later amendments, and registered on ClinicalTrials.gov (NCT05104619). The patients/participants provided their written informed consent to participate in this study. Written informed consent was obtained from the individual(s) for the publication of any potentially identifiable images or data included in this article.

## Author Contributions

NO, IF, EC, and RT wrote the original draft of the manuscript. NO, IF, CA, EC, ST, and SD designed the experiments. NO performed the experiments. EC, ST, and SD informed and included patients. RT and AB collected the SCC fresh-frozen and collected and sectioned the FFPE SCC sample. CA and RT performed the histology and validated diagnostics. NO and IF analyzed the data. NO, IF, and EC corrected the manuscript. IF, MS, ST, and SD supervised the project. IF and SD provided the funding. All authors contributed to the article and approved the submitted version.

## Funding

This study was funded by the Ministère de l'Enseignement Supérieur, the de la Recherche et de l'Innovation, the Cancéropôle Nord-Ouest AAP CNO Emergence 2020, and the Université de Lille and Inserm.

## Conflict of Interest

MS and IF are inventors of the patent (priority number WO2015IB57301 20150922) related to part of the described protocol. The remaining authors declare that the research was conducted in the absence of any commercial or financial relationships that could be construed as a potential conflict of interest.

## Publisher's Note

All claims expressed in this article are solely those of the authors and do not necessarily represent those of their affiliated organizations, or those of the publisher, the editors and the reviewers. Any product that may be evaluated in this article, or claim that may be made by its manufacturer, is not guaranteed or endorsed by the publisher.
