# Supplementary material for: Mass Spectrometry-Based Differentiation of Oral Tongue Squamous Cell Carcinoma and Nontumor Regions With the SpiderMass Technology
Source: Front Oral Health. 2022 Mar 3;3:827360. doi: 10.3389/froh.2022.827360 (PMC8929397; doi:10.3389/froh.2022.827360)
Supplement: Supplementary file 1 [file Data_Sheet_1.docx]

| 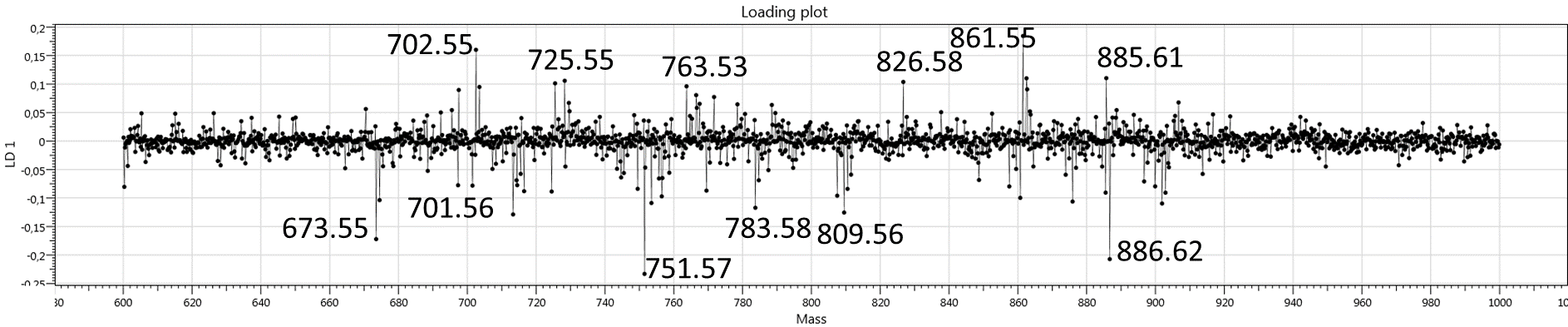 |
| --- |
| **Figure S1.** PCA-LDA mass loading plot of the LD1 from the FFPE databank. Several lipid ions were found more discriminative in the non-tumour (top) and the tumour (bottom) region. |

| 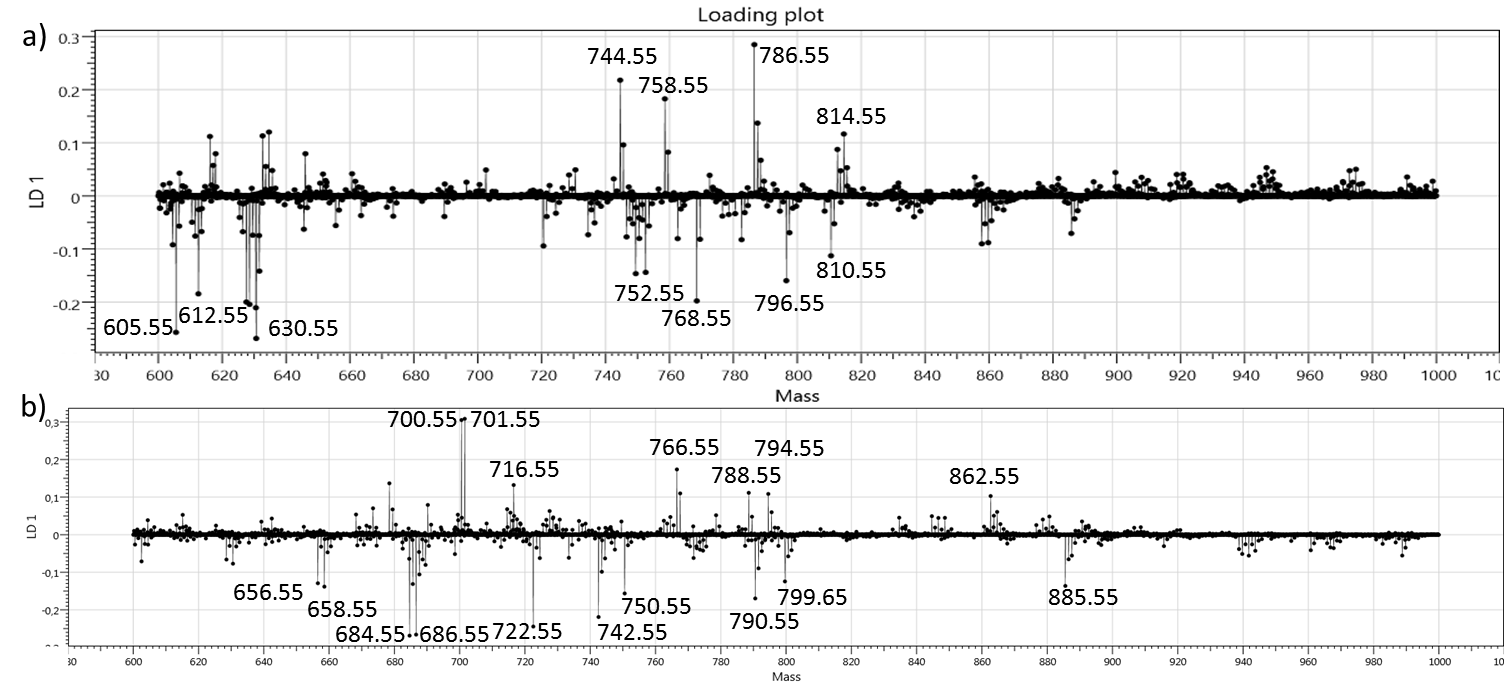 |
| --- |
| **Figure S2**. PCA-LDA mass loading plot of the LD1 from the prospective databank in the a) positive and b) negative ion mode. Several lipid ions were found more discriminative. In the positive ion mode the top part corresponds to the non-tumour region and the bottom to the tumour region. In the negative ion mode the top represents the tumour and the bottom the non-tumour region. |

**Table S1**. Tentative annotations of the discriminative molecular species from FFPE SCC tissues in negative ion mode.

| *m/z* ion  in negative ion mode | Tentative annotation |
| --- | --- |
| *m/z* 673.55 | DAG (40:3) [M-H]^-^ or  DAG (O-38:5) [M+HCOO]^-^ |
| *m/z* 701.56 | PA (36:1) [M-H]^-^ or SE (46:6) [M+HCOO]- or DAG (O-40:5) [M+HCOO]^-^ TAG (34:0;1) [M+CH_3_OCOO]^-^ |
| *m/z* 702.55 | PE (O-34:1) [M-H]^-^, HexCer 32:0;1 [M+HCOO]^-^ or Cer 38:0;5 [M+CH_3_COO]^-^ |
| *m/z* 725.55 | PA (38:3) [M-H]^-^ or SE (48:8) [M+HCOO]^-^ |
| *m/z* 751.57 | PA (40:4) [M-H]^-^, SM 32:1;4 [M+HCOO]^-^, or DAG (42:8) [M+CH_3_COO]^-^ or SE (50:9) [M+HCOO]^-^ |
| *m/z* 763.53 | TAG (42:9) [M+CH_3_COO]^-^ or DAG (44:9) [M+HCOO]^-^ |
| m/ z 783.58 | PA (42:2) [M-H]^-^ |
| *m/z* 809.56 | PI (32:0) [M-H]^-^ or TAG (46:7) [M+HCOO]^-^, SM 36:0;4 [M+HCOO]^-^ or PA (44:3) [M-H]^-^ |
| *m/z* 826.58 | PC (36:4) [M+HCOO-] |
| *m/z* 861.55 | PI (36:2) [M-H]^-^, TAG (50:16) [ M+CH_3_COO]^-^, TAG (48:10;1) [M+CH_3_COO]^-^ |
| *m/z* 885.61 | PI (20:4_18:0) [M-H]^-^ |

**Table S2**. Tentative annotations of the molecular species from fresh-frozen SCC tissues in positive and negative ion mode

| *m/z* ion  In positive ion mode | Tentative annotation |
| --- | --- |
| *m/z* 744.55 | PS-(P-16:0/18:2) [M+H]^+^ |
| *m/z* 752.55 | PE (0-18:0/20:4) [M+H]^+^ |
| *m/z* 758.55 | PC (34:2) [M+H]+ or PC (O-32:0) [M+K]+ |
| *m/z* 768.55 | PE (36:1) [M+Na]^+^ or PE (38:4) [M+H]^+^ |
| *m/z* 786.55 | PC (O-36:6) [M+Na]^+^ or PC (O-38:9) [M+H]^+^ or (PC O-34:0) [M+K]^+^ |
| m/ z 796.55 | PC (34:2) [M+K]^+^ or PE (O-40:8) [M+Na]^+^ |
| *m/z* 810.55 | PC (38:4) [M+H]^+^ |

| *m/z* ion  In negative ion mode | Tentative annotation |
| --- | --- |
| *m/z* 700.55 | PE (O-34:2) [M-H]^-^ or HexCer 34:0;2[M-H]^-^ |
| *m/z* 701.55 | PA(36:1) [M-H]^-^ |
| *m/z* 716.55 | PE (34 :1) [M-H]^-^ |
| *m/z* 722.55 | PE (P-36 :4) [M-H]^-^ |
| *m/z* 742.55 | PE (36 :2) [M-H]^-^ or PC (34:2) [M-CH_3_-H]^-^ |
| *m/z* 750.55 | PE (36:4) [M-H]^-^ or PE (P-38:4) [M-H]^-^ |
| *m/z* 766.55 | PS (P-16:0/20:4) [M-H]^-^  PE (18:0/20:4) [M-H]^-^  PC (16:0/20:4) [M-CH_3_-H]^-^ |
| *m/z* 788.55 | PS (36 :1) [M-H]^-^ |
| *m/z* 790.55 | PS 36:0 or CerP 44:0;4 [M-H]^-^ |
| *m/z* 794.55 | PC (38:4) [M-CH_3_-H]^-^ or PC (16:0/18:1) [M + Cl]^-^ |
| *m/z* 862.55 | SHexCer d18:1;22:0  PC(P-18:0_22:1))+Cl^-^ |
| *m/z* 885.55 | PI(20:4_18:0) [M-H]^-^ |
